# Carbonic anhydrase IX is a predictive marker of doxorubicin resistance in early-stage breast cancer independent of HER2 and TOP2A amplification

**DOI:** 10.1038/bjc.2012.32

**Published:** 2012-02-14

**Authors:** A S Betof, Z N Rabbani, M E Hardee, S J Kim, G Broadwater, R C Bentley, S A Snyder, Z Vujaskovic, E Oosterwijk, L N Harris, J K Horton, M W Dewhirst, K L Blackwell

**Affiliations:** 1 Department of Pathology, Duke University Medical Center, Room 201 MSRB, Research Drive, Box 3455 DUMC, Durham, NC, USA; 2 Department of Radiation Oncology, Duke University Medical Center, Room 201 MSRB, Research Drive, Box 3455 DUMC, Durham, NC, USA; 3 Department of Internal Medicine, Division of Oncology and Hematology, Korea University Medical Center, Seoul, South Korea; 4 Cancer Statistical Center, Duke Cancer Institute, Box 2717 DUMC, Durham, NC, USA; 5 Department of Experimental Urology, University Medical Center Nijmegen, Nijmegen, The Netherlands; 6 Department of Medicine, Division of Medical Oncology, Yale University, New Haven, CT, USA; 7 Department of Hematology/Oncology, Duke University Medical Center, Durham, NC, USA

**Keywords:** breast cancer, doxorubicin, CA IX, hypoxia

## Abstract

**Background:**

In early-stage breast cancer, adjuvant chemotherapy is associated with significant systemic toxicity with only a modest survival benefit. Therefore, there is considerable interest in identifying predictive markers of response to therapy. Doxorubicin, one of the most common drugs used to treat breast cancer, is an anthracycline chemotherapeutic agent, a class of drugs known to be affected by hypoxia. Accordingly, we examined whether expression of the endogenous hypoxia marker carbonic anhydrase IX (CA IX) is predictive of outcome in early-stage breast cancer patients treated with doxorubicin.

**Methods:**

We obtained 209 early-stage pre-treatment surgically-resected breast tumours from patients, who received doxorubicin in their chemotherapeutic regimen and had >10 years of follow-up. Immunohistochemistry was used to detect CA IX, and we used fluorescence *in situ* hybridisation to detect both human epidermal growth factor receptor (*HER2*) and DNA topoisomerase II-alpha (*TOP2A*) gene amplification.

**Results:**

Carbonic anhydrase IX intensity was significantly correlated with progression-free survival (PFS) and overall survival (OS) in patients receiving 300 mg m^−2^ of doxorubicin (HR=1.82 and 3.77; *P*=0.0014 and 0.010, respectively). There was a significant, inverse correlation between CA IX score and oestrogen receptor expression, but no significant correlations were seen with either HER2 or TOP2A ratio.

**Conclusion:**

We demonstrate that CA IX expression is correlated with worse PFS and OS for breast cancer patients treated with doxorubicin, independent of *HER2* or *TOP2A* gene amplification. This study provides evidence that using CA IX to detect hypoxia in surgically-resected breast tumours may be of clinical use in choosing an appropriate chemotherapy regimen.

Hypoxia, a pathological feature of many solid tumours, is caused by an imbalance between tumour proliferation and angiogenesis (Dang and Semenza, 1999; Dewhirst *et al*, 2008). Hypoxic regions are defined by an oxygen tension (pO_2_) of ⩽10 mm Hg. They have been identified in up to 50% of locally advanced breast tumours (Vaupel *et al*, 2002), and the presence of hypoxia is known to be a negative predictor of survival in cancer patients as it may contribute to more aggressive tumour phenotypes, increased invasiveness, and metastasis (Harris, 2002).

The transmembrane glycoprotein carbonic anhydrase IX (CA IX) has been identified as a potentially important marker of hypoxia in breast tumours. Known to catalyse the transformation of carbon dioxide to carbonic acid, CA IX may contribute to acidification of the extracellular microenvironment in a variety of tumours (Pastorek *et al*, 1994; Lindskog, 1997). Expression of CA IX is dependent on the transcription factor hypoxia-inducible factor-1 (HIF-1) (Wykoff *et al*, 2000), and the presence or absence of CA IX is correlated with microelectrode measurements of tumour oxygenation in cervical carcinoma (Loncaster *et al*, 2001). Immunohistochemical studies demonstrate that CA IX co-localises with pimonidazole, a bioreductive marker of hypoxia (Olive *et al*, 2001). Although CA IX is widely accepted as a marker of tumour hypoxia, its prognostic significance remains the subject of significant debate (Chia *et al*, 2001; Bartosova *et al*, 2002; Span *et al*, 2003; Brennan *et al*, 2006). The differing conclusions in these studies may be explained by the fact that heterogeneous patient populations have been treated with different combinations of surgery, radiation, and chemotherapy.

Breast cancer is the most common malignancy affecting women today aside from non-melanoma skin cancer, and it trails only lung cancer as the most common cause of cancer death in females (Jemal *et al*, 2008). Although standard surgical and radiotherapy techniques have resulted in over 95% local control of primary breast tumours (Bartelink *et al*, 2001), the 10-year overall survival (OS) rate still hovers just above 80% because of failures of local and systemic therapies (Navalta *et al*, 2010). Although surgery remains the mainstay of early-stage breast cancer treatment, approaches involving adjuvant chemotherapy are increasing. In early stage disease, adjuvant chemotherapy provides only a modest survival benefit while causing significant systemic toxicity and patient suffering. Thus, there is considerable interest in identifying predictive markers of response to chemotherapy to enable clinicians to select agents most likely to benefit a given patient and avoid ineffectual treatments.

Hypoxia is also widely accepted to have a role in resistance to radiotherapy and chemotherapy in a variety of human tumours. Anthracyclines, a group of chemotherapeutic agents that inhibit topoisomerase II*α*, are the most common chemotherapeutic agents used worldwide to treat breast cancer. Under hypoxic conditions, cancer cells experience a large pH gradient across the cellular membrane, maintaining acidic extracellular and basic intracellular environments (Svastova *et al*, 2004). The acidic extracellular environment can decrease the uptake of anthracyclines by cells, because these drugs are weak bases, which ionise at low pH (Gerweck and Seetharaman, 1996). Furthermore, one of the mechanisms by which anthracyclines mediate cellular death is through iron-mediated generation of reactive oxygen species (ROS). As ROS formation is dependent on the presence of oxygen within the tumour microenvironment, anthracyclines may be less effective in hypoxic tumours. To address this potential disparity in therapeutic effectiveness, we gathered a set of early-stage breast cancer biopsies from patients who received anthracycline-based chemotherapeutic regimens and had >10 years of follow-up. Using immunohistochemical detection of CA IX, we demonstrate that tumour hypoxia is correlated with worse outcome for early-stage breast cancer patients treated with doxorubicin. Further, we found CA IX to predict outcome, independent of human epidermal growth factor receptor 2 (*HER2*) and DNA topoisomerase II-alpha (*TOP2A*) gene amplification.

## Materials and methods

### Study population

Breast cancer tissue samples were obtained from 209 patients from the breast cancer tissue bank of the Specialized Program of Research Excellence (SPORE) at Duke University Medical Center, Durham, NC, USA. Patient records were assessed, and 500 patients were identified who had received doxorubicin-containing chemotherapy and had a minimum of 9 years of follow-up. Of these, 209 samples had adequate tissue for analysis and had been followed >10 years or until tumour recurrence occurred.

Surgically excised tissues were fixed in 10% formalin, embedded in paraffin, and processed for routine histopathology. Initial histopathological evaluation was performed by a senior pathologist (RCB) and diagnosis of breast cancer was verified before processing for immunohistochemistry. Oestrogen (ER) and progesterone (PR) receptor values were determined by ligand binding assay. Fluorescence *in situ* hybridisation (FISH) was used to detect amplifications in *HER2* and *TOP2A* (Pauletti *et al*, 1996). Established prognostic variables were available for a majority of the tumours, including lymph nodes status, tumour size, ER status, and PR status. The clinicopathological characteristics of the entire cohort are summarised in Table 1.

### Treatment regimen

All patients were treated at the Duke University Medical Center, Durham, NC, USA, and underwent either modified radical mastectomy or lumpectomy with or without breast irradiation. Two adjuvant chemotherapy regimens were used: cyclophosphamide/adriamycin (doxorubicin)/5-fluorouracil (CAF) and cyclophosphamide/adriamycin (CA). The majority of patients (*n*=149, 71%) received four courses of CAF (cumulative dose of doxorubicin=300 mg m^−2^), whereas the other patients (*n*=60, 29%) received four cycles of AC (cumulative dose of doxorubicin=240 mg m^−2^).

### Carbonic anhydrase IX Immunohistochemistry

Immunohistochemistry for CA IX was performed with 5-*μ*m sections of formalin-fixed, paraffin-embedded tissues placed onto positively charged glass slides with a single-staining procedure. For the detection of CA IX, we used the anti-CA IX mouse monoclonal antibody clone M75 (from Dr Oosterwijk, Department of Urology, University Hospital Nijmegen, The Netherlands) (Zavada *et al*, 1993). Tissue sections were de-waxed and rehydrated. After quenching endogenous peroxidases with methanol and 3% hydrogen peroxide for 10 min, microwave antigen retrieval was done twice on high power for 5 min each in citrate buffer. After blocking with 10% donkey serum for 1 h, the slides were incubated with primary antibody (3 *μ*g ml^−1^) overnight at 4 °C and washed with Tris-buffered saline (TBS) (Pastorekova *et al*, 1992). A positive control was human non-small cell carcinoma tissue, which has previously been established as positive for CA IX (Kim *et al*, 2004, 2005). Simultaneous incubation of slides in which primary antibody was omitted served as a negative control. Biotinylated donkey anti-mouse antibody (1 : 1000, v/v) (Jackson ImmunoResearch Laboratories, Inc., West Grove, PA, USA) was applied for 30 min at room temperature, followed by application of avidin-biotin-peroxidase complex per the manufacturer's protocol (Vectastain ABC Kit, Vector Laboratories, Inc., Burlingame, CA, USA). Slides were again washed in TBS and colour was developed by a 5 min incubation in 3,3′-diaminobenzidine solution. Slides were counterstained with haematoxylin and mounted.

### Assessment of CA IX expression

The staining was performed by two investigators (ZNR and MEH) and slides were evaluated by a single board certified surgical pathologist with expertise in breast pathology (RCB), who was blinded to clinical outcome. Semi-quantitative interpretations of immunohistochemistry were performed as described (Chia *et al*, 2001). In brief, a score of 0–3 for the intensity of staining in the majority of the entire section with invasive carcinoma was given (CA IX intensity: 0=no staining; 1=weak; 2=moderate; 3=strong). All slides were evaluated by light microscopy and the percentage of tumour cells throughout the section that were stained positively was estimated (% of CA IX). The product of the intensity staining and the percentage of tumour produced a final immunostaining score (CA IX score) of 0–300.

### Statistical methods

Summary statistics were reported for the baseline discrete as well as continuous variables. Spearman's correlation coefficients were used to study the pair-wise association between HER2, TOP2A, ER expression, and PR expression, separately with CA IX measurements. The proportion of CA IX highly-positive samples, which were negative for ER and PR expression were compared using the Pearson's test of proportions. Overall survival was measured from the time of diagnosis to death and was censored at last follow-up if death did not occur. Progression-free survival (PFS) was measured from the time of diagnosis to recurrence or death and was censored for patients without an event at last follow-up date. Kaplan–Meier estimated survival curves were used to display the OS and PFS and by CA IX status. Curves were compared using the log-rank test. Cox proportional hazards regression models were used to determine if one or more variables, including CA IX measurements, type of treatment, patient baseline characteristics, and disease statuses, were predictive of overall OS or PFS.

## Results

### Patient characteristics

The clinicopathological features of this cohort of 209 women with invasive breast cancer are described in detail in Table 1. The median age was 48 (range 24–79) years, the median tumour size was 3 cm (range 0.3–12), 86% had node-positive disease, 53% were positive for ER, and 49% positive for PR. Of the 206 patients for whom recurrence data are available, 130 experienced recurrence and 76 did not. The median duration of follow-up for the entire cohort was 8.3 (range 0.2–20.1) years. All patients were treated with appropriate surgical therapy and had negative surgical margins. Compared with other similar studies, this cohort had the advantage of all patients receiving anthracycline-based chemotherapy regimens; previous studies have largely considered heterogeneous patient groups where a variety of treatment modalities are employed. Furthermore, we have extensive follow-up data on these patients, and ER/PR status is predictive of clinical outcome (PFS based on ER/PR status, either positive HR=0.60, *P*=0.010), as we would expect. As with all clinical cohorts, our data set is somewhat limited by the number of available patients. Another limitation is that two different doses of doxorubicin were administered.

### Predicted outcome based on HER2

Table 2 displays the results of univariate Cox proportional hazards regression modelling for predicting PFS and OS based on FISH measurements of HER2. Univariately, mean HER2 is predictive of worse PFS (HR=1.65, *P*=0.038), further validating our data set.

### Carbonic anhydrase IX intensity, % of positive tumour cells, and score

**Figure 1:**
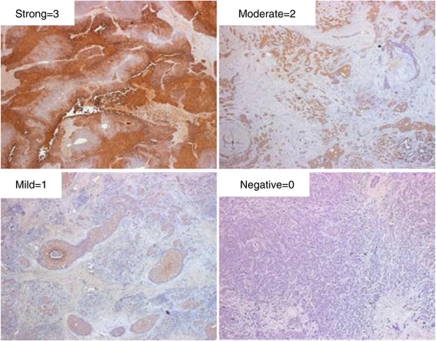
Examples of CA IX immunohistochemical staining. Carbonic anhydrase IX expression was evaluated by analysing the CA IX intensity, percent of positive tumour cells, and CA IX score. Cases were classified as positive if the overall CA IX score was greater than or equal to 50. Of the 209 cases analysed, 182 (87%) cases were positive for CA IX expression with both membranous and cytoplasmic localisation. The average intensity of CA IX staining was 1.44 on a scale of 0–3. The average percentage of tumour cell positively stained with CA IX was 68% and the mean CA IX score was 94.

Carbonic anhydrase IX expression was evaluated by analysing the CA IX intensity, % of positive tumour cells, and CA IX score. Figure 1 shows examples of the CA IX staining. Cases were classified as positive if the overall CA IX score was ⩾50. Of the 207 cases analysed, 182 (88%) cases were positive for CA IX expression, which is in line with previous reports (Span *et al*, 2003; Brennan *et al*, 2006). The staining pattern was characteristic of membranous and cytoplasmic localisation. The average intensity of CA IX staining was 1.44 on a scale of 0–3. The average percentage of tumour cells positively stained with CA IX was 68% and the mean CA IX score was 94.

### Association of CA IX expression with hormone receptor status

We evaluated the relationship between hormone receptor expression and CA IX staining to lend insight to conflicting reports in the literature regarding such a connection. We found a significant inverse correlation between ER expression and CA IX expression (*P*<0.01). No such correlation was observed between PR expression and CA IX (*P*=0.2). Of note, when the samples were grouped according to staining intensity (intensity score ⩽3), a significantly larger proportion of the CA IX highly-positive samples were negative for ER and PR expression (*P*=0.015).

### Association of CA IX expression with adjuvant therapy outcomes

**Figure 2:**
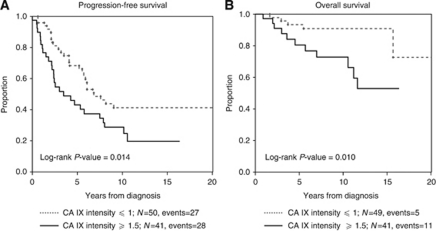
Kaplan–Meier survival curves for patients treated with CAF chemotherapy. Carbonic anhydrase IX staining intensity was significantly predicts (**A**) PFS (*P*=0.014) and (**B**) OS (*P*=0.010).

Two adjuvant chemotherapy regimens were used in this patient series: CAF and CA. The dose of doxorubicin in the CAF regimen was 300 mg m^−2^, whereas patients who received the CA regimen (*n*=60) received 240 mg m^−2^. When patient outcomes were evaluated based on the adjuvant chemotherapy regimen, CA IX intensity significantly predicted PFS and OS in the patients treated with CAF chemotherapy (HR=1.82 and 3.77; *P*=0.014 and 0.010, *n*=91 and 90, respectively). The Kaplan–Meier analyses revealing differential PFS and OS in the CAF group are shown in Figure 2. At the lower dose of doxorubicin, no significant relationship between CA IX intensity and PFS was observed. In addition, both OS and PFS were significantly lower in the CAF-treated patients with ER- and PR-negative tumours than those whose tumours were positive for either or both receptor (*P*<0.001).

### Carbonic anhydrase IX score is independent of HER2 and TOP2A amplification

**Figure 3:**
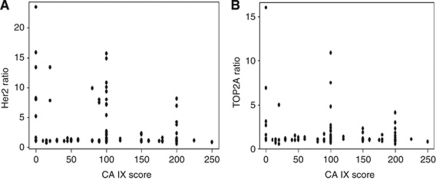
We calculated Spearman's correlation coefficients to determine the association of CA IX score with known predictive indicators such as HER2 and TOP2A amplification. Using FISH to detect both HER2 and TOP2A, we calculated the HER2 ratio and TOP2A ratio (number of genes of interest/number of chromosome 17) to identify gene amplification. Here we show the relationship between the CA IX score and HER2 ratio (**A**) and TOP2A ratio (**B**). There is no significant relationship between CA IX score and either HER2 or TOP2A ratio (*n*=140; Rho=−0.031 and 0.023, *P*=0.72 and 0.78, respectively).

We calculated Spearman's correlation coefficients to determine the association of CA IX score with known predictive indicators such as *HER2* and *TOP2A* gene amplification. Using FISH to detect both *HER2* and *TOP2A*, we calculated the HER2 ratio and TOP2A ratio (number of genes of interest/number of chromosome 17) to identify gene amplification in our samples. Figure 3 shows scatter plots of the relationship between CA IX score and these two predictive variables. No significant correlation is seen between CA IX score and either HER2 ratio or TOP2A ratio (*n*=140). Thus, CA IX is a predictive marker of outcome in early-stage breast cancer patients treated with doxorubicin independent of *HER2* and *TOP2A* amplification.

## Discussion

Doxorubicin is one of the most active chemotherapeutic agents for the treatment of advanced breast cancer (Honig, 1996; Lindley, 1997; Ranson *et al*, 1997; Winer *et al*, 2001). However, not all patients benefit from doxorubicin and its anti-tumour benefits are counterbalanced by significant toxicities (Hurny *et al*, 1996; Doyle *et al*, 2005; Hershman *et al*, 2007; Pinder *et al*, 2007). The most noteworthy doxorubicin toxicity is cardiomyopathy, but this agent is also associated with haematological toxicity, secondary leukaemia, and hepatic impairment (2010). Given the high rate of resistance to doxorubicin, coupled with the severe toxicities associated with its use, the choice of whether or not to use this effective anti-tumour agent in a particular patient is challenging. Accordingly, a predictive indicator of response to doxorubicin would be a valuable tool for clinical decision making.

Our results indicate that the endogenous hypoxia marker CA IX could be used to predict doxorubicin treatment efficacy; high expression of CA IX is correlated with worse outcomes in patients treated with CAF chemotherapy. Carbonic anhydrase IX has received considerable attention as a marker of hypoxia and indicator of patient prognosis. Previous studies report that high expression of CA IX is correlated with worse outcomes in a variety of cancers, including breast cancer (Koukourakis *et al*, 2001; Loncaster *et al*, 2001; Hui *et al*, 2002; Swinson *et al*, 2003; Bui *et al*, 2004; Hussain *et al*, 2004). Furthermore, expression of CA IX has been shown to predict disease-free survival and response to epirubicin in hormone-responsive breast cancer patients treated with neoadjuvant epirubicin followed by adjuvant cyclophosphamide, methotrexate, and 5-fluorouracil (Generali *et al*, 2006). Epirubicin is a doxorubicin analogue that functions by similar mechanisms, so we hypothesised that CA IX would also be predictive of response to doxorubicin. Indeed, this study is the first to show that expression of CA IX is predictive of CAF chemotherapeutic effectiveness, suggesting that patients whose tumours strongly express CA IX should be treated with a non-anthracycline containing chemotherapeutic regimen. Doing so could spare these patients from a likely ineffective treatment with a highly toxic agent.

This stratification approach to treatment selection may be particularly effective for patients with more aggressive cancer subtypes. Patients with ER negative tumours have significantly worse outcomes regardless of tumour stage, so appropriate and tailored chemotherapeutic selection is especially critical in this population (Ries and Eisner, 2007). As in other reports, CA IX expression in our series correlated significantly with negative ER status (Tan *et al*, 2009). This key steroid receptor may, in fact, be downregulated by hypoxia (Cooper *et al*, 2004). Furthermore, beyond transcriptional repression of ER, proliferation could be driving high levels of oxygen consumption leading to hypoxia. The oxygen consumption rate of proliferating cells is three to five times higher than quiescent cells (Freyer *et al*, 1984). Accordingly, it has been shown that oxygen consumption rate is five to thirty times more influential in causing hypoxia than alterations in perfusion or vascular oxygen concentration (Secomb *et al*, 1995). As most highly proliferative cells do not express ER, oxygen consumption could explain the negative relationship between expression of CA IX and ER (Jensen *et al*, 2001). In this high-risk patient population, evidence-guided selection of chemotherapeutic agents alongside of strategies to inhibit cell signalling pathways activated by hypoxia might improve treatment efficacy and disease outcomes.

In this series, both radiation and 5-FU are potential confounding factors because of differences in treatment regimens. However, similar proportions of patients with high- and low-CA IX staining intensity received radiation therapy (*P=*0.19), so radiation therapy is unlikely to explain the differences in PFS and OS that correlate with differential expression of CA IX. It has been reported that hypoxia can reduce the cytotoxicity of 5-FU (Grau and Overgaard, 1992). This may occur by the initiation of cell-cycle arrest and/or by inhibiting activation of p53 signalling pathways (Achison and Hupp, 2003; Yoshiba *et al*, 2009). Thus, we cannot rule out that differences in outcome for the group of patients treated with CAF could have been influenced by the addition of 5-FU to the treatment regimen.

The identification of CA IX as an important marker of doxorubicin-treatment outcome is strengthened by our finding that this marker has predictive power independent of *HER2* and *TOP2A* gene amplification status. Several large clinical studies conducted by multiple groups indicate that anthracycline-containing regimens only incrementally benefit the ∼25% of breast cancer patients that have HER2 amplification or overexpression over non-anthracycline-containing regimens (Muss *et al*, 1994; Paik *et al*, 1998; Thor *et al*, 1998; Dressler *et al*, 2005; Piccart-Gebhart, 2006; Pritchard *et al*, 2006; Gennari *et al*, 2008). Furthermore, the *HER2* gene is located on chromosome 17q21-22, close to *TOP2A*, resulting in 33–60% of *HER2*-positive tumours containing concurrent modifications of *TOP2A* (Smith *et al*, 1993; Jarvinen *et al*, 2000; Bofin *et al*, 2003; Olsen *et al*, 2004). Anthracyclines, including doxorubicin, inhibit topoisomerase II*α*, the protein product of *TOP2A*, which is an essential mediator of DNA replication and RNA transcription (Withoff *et al*, 1996b). So, it was unsurprising when overexpression of TOP2A protein was correlated with increased sensitivity to anthracyclines (Withoff *et al*, 1996a, 1996b; Jarvinen *et al*, 2000).

In our series, the value of CA IX as a predictive marker of response to doxorubicin appears to be independent of *HER2* and/or *TOP2A* amplification, suggesting a novel mechanism of anthracycline treatment resistance related to tumour hypoxia. One of the key mechanisms of anthracycline cytotoxicity is the drug's ability to reduce molecular oxygen to oxygen free radicals, and its effectiveness has been shown to be diminished both *in vitro* and *in vivo* in response to reduced availability of oxygen (Davies and Doroshow, 1986; Doroshow and Davies, 1986; Teicher *et al*, 1990; Frederiksen *et al*, 2003; Song *et al*, 2006). There are very few predictive markers available for sensitivity to anthracycline therapy and therapeutic interventions that could modulate hypoxia should have an important role in improving anthracycline sensitivity. Numerous strategies are currently under investigation to directly target tumour hypoxia, including the hypoxia cytotoxin tirapazamine, HIF-1 targeted agents, gene therapy, and increasing oxygenation using erythropoietin (Shannon *et al*, 2003). However, the success of these hypoxia targeting strategies has been limited to date. Given the high rate of breast tumour hypoxia identified in our primary breast cancer series, further investigation is warranted to explore the mechanisms underlying hypoxia-related doxorubicin resistance.

## Figures and Tables

**Table 1:** Patients’ clinicopathological characteristics

**Characteristics**	**Number of patients**	**Percentage (%)**
Total patients	209	
		
*Age, (median, range) years (*n*=206)*	48 (24–79)	
<50	119	57.8
⩾50	87	42.2
		
*Surgical treatment (*n*=199)*		
Lumpectomy	8	4.0
Lumpectomy+RT	25	12.6
Mastectomy	120	60.3
Mastectomy+RT	46	23.1
		
*Tumour size, (median, range) cm (*n*=194)*	3 (0.3–12)	
⩽2	61	31.4
>2–5	105	54.1
>5	28	14.4
		
*Node status (*n*=196)*		
Negative (0)	27	13.8
Positive (⩾1)	169	86.2
		
*ER/PR status (*n*=188)*		
Both negative	71	37.8
Either positive	117	62.2
		
*HER2 status (*n*=142)*		
FISH ratio <2.2	114	80.3
FISH ratio ⩾2.2	28	19.7
		
*TOP2A status (*n*=142)*		
FISH ratio <2.0	126	88.7
FISH ratio ⩾2.0 (amplification)	16	11.3
		
*Adjuvant therapy (*n*=201)*		
Chemotherapy *(doxorubicin)*		
240 mg m^−2^	81	40.3
300 mg m^−2^	120	59.7
		
*Recurrence type (*n*=206)*		
None	76	36.9
Local	14	6.8
Regional	24	11.7
Distant	75	36.4
Other	17	8.2
		
*Follow-up, (median, range) years (*n*=193)*	8.3 (0.2–20.1)	
No recurrence	72	37.3
Recurrence	89	46.1
Death (recurrence followed by death, death)	32	16.6

Abbreviations: ER=oestrogen receptor; FISH=fluorescence *in situ* hybridisation; HER2=human epidermal growth factor receptor 2; PR=progesterone receptor; RT=radiation therapy; TOP2A=DNA topoisomerase II-alpha.

**Table 2:** Univariate prediction of progression-free survival and overall survival based on *HER2* and *TOP2A* amplification

	**Progression-free survival**		**Overall survival**	
**Variable**	**Hazard ratio**	***P*-value**	**Hazard ratio**	***P*-value**
*HER2* mean (continuous)	1.03	0.069	1.06	0.043
*HER2* mean (<4/⩾4)	1.65	0.038	1.73	0.23
*TOP2A* mean (continuous)	1.09	0.011	1.14	0.024
*TOP2A* mean (<4/⩾4)	2.32	0.0027	2.59	0.065

Abbreviations: *HER2*=human epidermal growth factor receptor 2; *TOP2A*=DNA topoisomerase II-alpha.

Univariately, mean *HER2* and mean *TOP2A* values ⩾4.0 are predictive of worse progression-free survival.
